# Prevalence and management of cardiovascular risk factors in Portuguese living in Portugal and Portuguese who migrated to Switzerland

**DOI:** 10.1186/s12889-015-1659-8

**Published:** 2015-03-31

**Authors:** Luís Alves, Ana Azevedo, Henrique Barros, Peter Vollenweider, Gérard Waeber, Pedro Marques-Vidal

**Affiliations:** EPIUnit - Institute of Public Health of the University of Porto (ISPUP), Porto, Portugal; Department of Clinical Epidemiology, Predictive Medicine and Public Health, University of Porto Medical School, Porto, Portugal; St. André de Canidelo Family Health Unit, Vila Nova de Gaia, Portugal; Department of Medicine, Internal Medicine, CHUV and Faculty of biology and medicine, Lausanne, Switzerland; Institute of Social and Preventive Medicine (IUMSP), Lausanne University Hospital, Biopole 2, Route de la Corniche 10, CH 1010 Lausanne, Switzerland

**Keywords:** Migration, Epidemiology, Risk factors, Diagnosis, Cardiovascular diseases

## Abstract

**Background:**

Information regarding the health status of migrants compared to subjects who remain in the country of origin is scarce. We compared the levels and management of the main cardiovascular risk factors between Portuguese living in Porto (Portugal) and Portuguese migrants living in Lausanne (Switzerland).

**Methods:**

Cross-sectional studies conducted in Porto (EPIPorto, 1999 to 2003, n = 1150) and Lausanne (CoLaus, 2003 to 2006, n = 388) among subjects aged 35–65 years. Educational level, medical history and time since migration were collected using structured questionnaires. Body mass index, blood pressure, cholesterol and glucose levels were measured using standardized procedures.

**Results:**

Portuguese living in Lausanne were younger, more frequently male and had lower education than Portuguese living in Porto. After multivariate adjustment using Poisson regression, no differences were found between Portuguese living in Porto or in Lausanne: prevalence rate ratio (PRR) and (95% confidence interval) for Portuguese living in Lausanne relative to Portuguese living in Porto: 0.92 (0.71 - 1.18) for current smoking; 0.78 (0.59 - 1.04) for obesity; 0.81 (0.62 - 1.05) for abdominal obesity; 0.82 (0.64 - 1.06) for hypertension; 0.88 (0.75 - 1.04) for hypercholesterolemia and 0.92 (0.49 - 1.73) for diabetes. Treatment and control rates for hypercholesterolemia were higher among Portuguese living in Lausanne: PRR = 1.91 (1.15 - 3.19) and 3.98 (1.59 - 9.99) for treatment and control, respectively. Conversely, no differences were found regarding hypertension treatment and control rates: PRR = 0.98 (0.66 - 1.46) and 0.97 (0.49 - 1.91), respectively, and for treatment rates of diabetes: PRR = 1.51 (0.70 - 3.25).

**Conclusions:**

Portuguese living in Lausanne, Switzerland, present a similar cardiovascular risk profile but tend to be better managed regarding hypercholesterolemia than Portuguese living in Porto, Portugal.

**Electronic supplementary material:**

The online version of this article (doi:10.1186/s12889-015-1659-8) contains supplementary material, which is available to authorized users.

## Background

Between 1990 and 2013, the number of international migrants in Europe increased from 50 to 72 million [1]. Migrants are generally seen as a vulnerable population with disadvantaged cardiovascular health profiles [2], although this statement has been challenged [3]. Indeed, cardiovascular risk varies according to country of origin [4], and some migrant groups have a lower cardiovascular risk than the general population of the host country [5-7]. Conflicting results regarding the management of cardiovascular risk factors among migrants have also been reported, some studies suggesting that migrants are less well managed than nationals from the host country [8,9], while other studies found no such differences [10,11]. Still, most migrant studies compared migrants and nationals from the host country [2], while data comparing migrants with the population of their country of origin are scarce [12,13]. Further, most studies addressed the effect of migration on subjects going from non-Western countries to Western countries, and less is known regarding migrants going from a Western country to another.

Although several studies have compared the health status of Portuguese migrants with Swiss nationals [14,15], little is known on how cardiovascular risk profiles and management differ between resident and migrant Portuguese. In a previous study [16], we showed that Portuguese migrants in Switzerland did not differ from resident Portuguese regarding most cardiovascular risk factors, but the study relied on self-reported rather than objectively measured data.

Thus, we aimed to compare the prevalence and management of the main cardiovascular risk factors between Portuguese living in Porto and Portuguese migrants living in Lausanne using objective data from two population-based studies. Our main hypothesis was that migrating to a wealthier country would translate into a better management of cardiovascular risk factors.

## Methods

### Sampling procedure

The methodologies of the EPIPorto and CoLaus studies have been described previously [17,18] and are summarized in Additional file 1: Table S1. Both studies have been approved by the local ethics committees and comply with the declaration of Helsinki. The CoLaus study was approved by the Commission cantonale d'Éthique de la Recherche sur l'être humain of canton Vaud, Lausanne, Switzerland (www.cer-vd.ch) and the EpiPorto was approved by the Comissão de Ética para a Saúde do Centro Hospitalar de São João e da Faculdade de Medicina da Universidade do Porto (file 65/95). All participants provided their written informed consent. As the prevalence of most cardiovascular risk factors is lower in younger individuals and most migrants tend to return to their original country after retiring, subjects aged <35 or >65 years (age for retirement in Switzerland) were excluded.

### Data collection

Data collection is summarized in Additional file 1: Table S2. In both studies, serum glucose level was determined using routine enzymatic methods; total and HDL cholesterol and triglyceride levels were determined using standard enzymatic colorimetric methods and low density lipoprotein cholesterol (LDL-C) levels were calculated using the Friedewald equation if triglycerides were <4.6 mmol/l.

In both studies, information on sociodemographic characteristics and medical history was collected by trained interviewers using structured questionnaires. Education was grouped as elementary, secondary or university. Ongoing treatment for hypertension, dyslipidemia and diabetes mellitus was considered according to reported information. Specifically in the CoLaus study information on length of stay in Switzerland was collected and dichotomized using 5 years as a cut-off value. Overweight was defined as a body mass index (BMI) ≥25 and <30 kg/m^2^; obesity for a BMI ≥ 30 kg/m^2^. Abdominal obesity was defined as waist > 102 cm (men) or >88 cm (women) [19]. Hypertension was considered if the participant had a systolic blood pressure (SBP) ≥140 mmHg or a diastolic blood pressure (DBP) ≥90 mmHg or was taking antihypertensive drugs. Treated hypertensive participants were considered controlled if they had SBP < 140 and DBP < 90 mm Hg. Hypercholesterolemia was considered if the participant had a total cholesterol > 5.2 mmol/L or was on hypolipidemic drugs. Treated hypercholesterolemic participants were considered controlled if they had a total cholesterol ≤ 5.2 mmol/L. Diabetes was considered if the participant had a glucose level ≥7.0 mmol/L or reported taking antidiabetic drugs.

### Statistical analysis

Statistical analyses were performed using Stata version 12.0 for Windows (Stata Corp, College Station, Texas, USA). Descriptive results were expressed as number of participants (percentage) or as average ± standard deviation. Bivariate analyses were performed using chi-square or Fisher’s exact test for qualitative variables and Student’s t-test for quantitative variables. For multivariate analysis of qualitative variables, Poisson regression was preferred to logistic regression because most outcomes of interest exceeded 20%. Results were expressed as prevalence rate ratio and 95% confidence interval. Statistical significance was assessed for p < 0.05.

## Results

### Characteristics of participants

The main characteristics of participants according to study are summarized in Table 1. Portuguese living in Lausanne were more frequently men, were younger and had a lower educational level than Portuguese living in Porto. The average length of stay for the Portuguese migrants was 18 ± 6 years (range 1 – 52), and 381 out of 388 (98.2%) had a length of stay ≥5 years.

**Table 1:** **Characteristics of Portuguese according to place of living**

	**Porto**	**Lausanne**	**p-value**
Sample size	1550	388	
Women (%)	972 (62.7)	175 (45.1)	<0.001
Age (years)	50.8 ± 8.2	44.2 ± 5.7	<0.001
Educational level			
Elementary	704 (45.5)	348 (89.9)	
Secondary	443 (28.6)	32 (8.3)	<0.001
University	401 (25.9)	7 (1.8)	

Results are expressed as number of participants (percentage) or as mean ± standard deviation. Statistical analysis by chi-square test or Student’s t-test.

### Prevalence and management of cardiovascular risk factors

The prevalence of cardiovascular risk factors according to place of living and the results of the multivariate analyses are summarized in Table 2. Portuguese living in Lausanne had a higher prevalence rate of being treated or controlled for hypercholesterolemia (Table 2). No differences were found regarding smoking; overall or abdominal obesity; hypertension prevalence, management and control; hypercholesterolemia prevalence; diabetes prevalence or treatment (Table 2), and similar findings were obtained after excluding subjects living in Switzerland for less than 5 years (Table 3).

**Table 2:** **Multivariate analysis of prevalence and management of cardiovascular risk factors in Portuguese migrants relative to Portuguese who remained in Portugal**

	**Porto**	**Lausanne**	**Prevalence rate ratio**	**p-value**	**Adjusting for gender, age, educational level and**
Current smoking	391 (25.5)	110 (28.4)	0.92 (0.71 - 1.18)	0.49	None
Anthropometry					
Obesity	358 (23.4)	74 (19.1)	0.78 (0.59 - 1.04)	0.09	Smoking
Abdominal obesity	475 (30.6)	81 (20.9)	0.81 (0.62 - 1.05)	0.11	Smoking
Blood pressure					
Hypertension	613 (39.6)	91 (23.5)	0.82 (0.64 - 1.06)	0.13	Obesity; abdominal obesity
Treated	320 (52.2)	33 (36.3)	0.98 (0.66 - 1.46)	0.93	Obesity; abdominal obesity
Controlled	107 (33.4)	12 (36.4)	0.97 (0.49 - 1.91)	0.93	Obesity; abdominal obesity
Blood lipids					
Hypercholesterolemia	1119 (72.2)	228 (58.8)	0.88 (0.75 - 1.04)	0.14	Obesity; abdominal obesity
Treated	128 (11.4)	26 (11.4)	1.91 (1.15 - 3.19)	0.01	Obesity; abdominal obesity
Controlled	35 (27.3)	16 (61.5)	3.98 (1.59 - 9.99)	0.003	Obesity; abdominal obesity
Glycemic status					
Diabetes	93 (6.0)	15 (3.9)	0.92 (0.49 - 1.73)	0.80	Obesity; abdominal obesity
Treated	64 (68.8)	11 (73.3)	1.51 (0.70 - 3.25)	0.30	Obesity; abdominal obesity

Results are expressed as number of participants (percentage) and as prevalence rate ratio and (95% confidence interval) of Portuguese migrants versus Portuguese who remained in Portugal. Statistical analysis by Poisson regression adjusting for gender, age, educational level and other covariates as indicated.

**Table 3:** **Multivariate analysis of prevalence and management of cardiovascular risk factors in Portuguese migrants relative to Portuguese who remained in Portugal**

	**Prevalence rate ratio**	**p-value**	**Adjusting for gender, age, educational level and**
Current smoking	0.92 (0.71 - 1.19)	0.53	None
Anthropometry			
Obesity	0.78 (0.59 - 1.04)	0.10	Smoking
Abdominal obesity	0.81 (0.62 - 1.06)	0.12	Smoking
Blood pressure			
Hypertension	0.82 (0.64 - 1.06)	0.12	Obesity; abdominal obesity
Treated	1.00 (0.67 - 1.48)	0.99	Obesity; abdominal obesity
Controlled	0.97 (0.49 - 1.91)	0.93	Obesity; abdominal obesity
Blood lipids			
Hypercholesterolemia	0.88 (0.74 - 1.04)	0.12	Obesity; abdominal obesity
Treated	1.96 (1.18 - 3.27)	0.01	Obesity; abdominal obesity
Controlled	3.98 (1.59 - 9.99)	0.003	Obesity; abdominal obesity
Glycemic status			
Diabetes	0.93 (0.5 - 1.76)	0.83	Obesity; abdominal obesity
Treated	1.51 (0.7 - 3.25)	0.30	Obesity; abdominal obesity

Results are expressed as prevalence rate ratio and (95% confidence interval) of Portuguese migrants versus Portuguese who remained in Portugal. Statistical analysis by Poisson regression adjusting for gender, age, educational level and other covariates as indicated.

Portuguese migrants living in Switzerland for less than 5 years were excluded.

## Discussion

To our knowledge, this is one of the few studies assessing differences in cardiovascular risk factor levels and management among migrants going from a developed country to another. Our results suggest that Portuguese migrants living in Lausanne, Switzerland, have a similar cardiovascular risk profile but are better managed regarding hypercholesterolemia than Portuguese living in Porto, Portugal.

### Characteristics of participants

In agreement with previous reports [20], Portuguese living in Lausanne were more frequently male, were younger and had a lower educational level than Portuguese living in Porto. These differences can be explained by the fact that unemployment rates in Portugal are higher among younger and/or lower educated people [21], urging them to migrate in search of an employment. The higher prevalence of men among Portuguese migrants can be explained by the fact that in the majority of Portuguese couples, men migrate first in search of employment, the wife joining afterwards.

### Prevalence and management of cardiovascular risk factors

Both Portugal and Switzerland have universal health insurance coverage, but the degree of coverage varies. In Switzerland, health insurance is compulsory and insurance premiums are paid independently of income. The insured person pays part of the cost of treatment. Between 1999 and 2010, health insurance premiums increased by 77%, coupled with increasing out-of-pocket payments [22,23]. In Portugal, the health service is based on a universal system access to medical care, comprehensive and free, funded by taxation and complemented by public insurance funds and private and direct payments [24]. Contrary to Switzerland, where the choice of a doctor is free and multiple doctors are allowed, in Portugal, the choice the doctor is not allowed to patients, the waiting time before being received by a doctor is sometimes long, which is troublesome during a sudden illness [25,26]. Hence, obtaining an appointment might be easier in Switzerland than in Portugal, thus increasing the likelihood of diagnosing and adequately managing cardiovascular risk factors. Indeed, more than 90% of the Portuguese migrants in Switzerland have a personal doctor and adopt a more preventive behavior than the other migrant populations, which translates into a higher propensity to frequently change doctor and challenge the diagnoses proposed [20].

Contrary to a previous study that compared self-reported data [16], no differences were found between Portuguese migrants and Portuguese living in Porto regarding obesity levels. Similarly, no differences were found regarding the prevalence of hypertension, hypercholesterolemia and diabetes, suggesting that migration to Switzerland does not impact the prevalence of cardiovascular risk factors among Portuguese. These findings go against the “health migrant effect”, i.e. that subjects who migrate tend to be healthier than subjects who remained in their country or even healthier than natives of the host country [27]. Possible explanations include the high unemployment rate and hard living conditions in Portugal, which would have prompted unemployed subjects to migrate irrespective of their health condition; another explanation is that Portuguese migrants tend to live together and maintain their original dietary habits, which would sustain the prevalence of cardiovascular risk factors as in their country of origin. Previous [28] and on-going studies comparing the lifestyle behaviors (i.e. physical activity and diet) of Portuguese migrants, Portuguese residents and Swiss nationals will help a better assessment of the determinants of cardiovascular risk factors among Portuguese migrants.

Sixty-two out of the 74 obese Portuguese migrants in Switzerland (84%) indicated having been told by their doctor that they had excess weight, a value considerably higher than the 17% previously reported for Portugal [29]. Differences in awareness and management may also account for the the fact that hypercholesterolemic Portuguese subjects living in Porto were significantly less likely to be treated and controlled than their migrant counterparts. Although no significant differences were found regarding hypertension treatment and control, less than 4 out of 10 hypertensive participants were treated, and less than 4 out of 10 treated hypertensive participants were controlled. These values are lower than those previously reported in Switzerland [30], which may imply some degree of heterogeneity in hypertension management between Portuguese migrants in Lausanne and Swiss nationals. Portuguese migrants also tended to have a higher likelihood of being treated for diabetes than Portuguese residents, but no statistical significance was obtained due to the small sample size. Overall, our results suggest that Portuguese living in Lausanne have a similar cardiovascular risk factor profile and tend to be better managed regarding hypercholesterolemia than Portuguese living in Porto.

### Strengths and limitations

This study has several strengths: first, it used data from two population-based surveys and relied on objective standardized and validated measurements. Second, sensitivity analyses performed after excluding migrants living in Switzerland for less than 5 years did not change our main conclusions (Table 3). Hence, the differences between Portuguese residents and migrants might be attributable to changes in living environment rather than to unmeasured individual characteristics associated with migration.

This study has also several limitations worth acknowledging. First, our migrant sample includes individuals migrating from urban and rural areas, while the resident sample is mostly urban. Still, the rural exodus observed in Portugal in the 60s and 70s was directed to the two main urban centers, Porto and Lisbon [31] and approximately half of the Portuguese residents was born outside Porto (data not shown). These might reduce comparability issues regarding the urban or rural origin of the participants. Second, the fact that the interviews in CoLaus were conducted in French might have selected migrants with adequate knowledge of the language. Still, their socio-economic features are in agreement with other studies conducted among Portuguese migrants [20]. Third, the participation rate in the CoLaus study was relatively low, but comparable with the MONICA surveys conducted in Switzerland and in other countries [32]. This might have led to the selection of health-conscious subjects, which could partly explain the better management of lipid levels among Portuguese migrants. Fourth, the two studies were not conducted simultaneously, and new European guidelines for management of hypertension and for cardiovascular disease prevention were issued in 2003 [33,34], which could have changed cardiovascular risk factor management among Portuguese migrants. Still, Switzerland has its own guidelines regarding cardiovascular disease management, and it has been shown that Swiss doctors use guidelines from different sources in their clinical practice [35]. Thus, it is likely the impact of the European guidelines in cardiovascular disease management in Switzerland after 2003 might be modest. Finally, other factors might intervene regarding the prevalence and management of cardiovascular risk factors. Married subjects are more likely to attend health checks [36]; subjects with a lower income tend to forgo healthcare for economic reasons [22,23]. Unfortunately, no such data was available or collected using similar criteria in both studies. The ongoing follow-up of the CoLaus cohort will include more detailed familial and socio-economic data such as work history, income, and housing conditions (location, neighborhood, pollution and noise levels). Further, in Switzerland, regional differences in cardiovascular risk factor screening and management have been reported, so that migration to regions other than Vaud might lead to different results [37].

## Conclusion

Portuguese living in Lausanne present a similar cardiovascular risk profile but tend to be better managed regarding hypercholesterolemia than Portuguese living in Porto.

## Additional file

Additional file 1:
**Table S1.** sampling procedures of the EpiPorto and the CoLaus studies. **Table S2.** data collection of the EpiPorto and the CoLaus studies.
