# Supplementary material for: Prevalence and management of cardiovascular risk factors in Portuguese living in Portugal and Portuguese who migrated to Switzerland
Source: BMC Public Health. 2015 Mar 31;15:307. doi: 10.1186/s12889-015-1659-8 (PMC4392627; doi:10.1186/s12889-015-1659-8)
Supplement: Additional file 1: — Table S1. sampling procedures of the EpiPorto and the CoLaus studies. Table S2. data collection of the EpiPorto and the CoLaus studies. [file 12889_2015_1659_MOESM1_ESM.docx]

**Supplementary table 1<**: sampling procedures of the EpiPorto and the CoLaus studies

|  | EpiPorto | CoLaus |
| --- | --- | --- |
| Location, number of inhabitants | Portugal, 240,000 inhabitants | Switzerland, 180,000 inhabitants |
| Sampling period | 1999-2003 | 2003-2006 |
| Inclusion criteria | (a) written informed consent; (b) willingness to take part in the examination and to provide blood samples | (a) written informed consent; (b) willingness to take part in the examination and to provide blood samples; (c) aged between 35 and 65 years and (d) Caucasian origin |
| Sampling method | Random digit dialing of landline telephones | Non-stratified population random sample |
| Participation rate | 70% | 41% |
| Final sample size | 2,485 | 6188 |
| Used sample size (Portuguese aged 35-65) | 1,550 | 388 |

**Supplementary table 2**: data collection of the EpiPorto and the CoLaus studies

|  | EpiPorto | CoLaus |
| --- | --- | --- |
| Fasting period (hours) | ≥12 | ≥8 |
| Anthropometric measurements |  |  |
| Participant | Light clothing and barefoot, standing position | Light clothing and barefoot, standing position |
| Height | To the nearest centimeter using a wall stadiometer | To the nearest 0.5 centimeter using a Seca® gauge |
| Weight | To the nearest 0.1 kilogram using a digital scale | To the nearest 0.1 kilogram using a Seca® scale |
| Waist circumference | Measured midway between the lower limit of the rib cage and the iliac crest using a flexible and non-distensible tape. Precision to the nearest centimeter | Measured at the narrowest point between the lowest rib and the iliac crest using a flexible and non-distensible tape. Average of two measurements |
| Blood pressure |  |  |
| Participant | Resting for 10 minutes | Resting for 10 minutes in the seated position |
| Device | Standard mercury sphygmomanometer | Omron® HEM-907 automated oscillometric sphygmomanometer with an adequately sized cuff |
| Number of measurements | Two. If the two readings differed more than 5 mm Hg, a third reading was taken | Three |
| Values reported | Mean (if two) or mean of the two closest readings | Mean of the last two readings |
